# Identification and Validation of Fibroblast‐Associated Genes in Osteoarthritis Based on High‐Dimensional Weighted Gene Coexpression Network Analysis

**DOI:** 10.1155/jimr/5547701

**Published:** 2025-09-28

**Authors:** Juan Xiao, Wei Lei, Hao Zhang, Feng Niu, Qunhai Wu, Honglin Pi, Poorani Gurumallesh

**Affiliations:** ^1^ Department of Orthopedics, Xiangyang Hospital of Traditional Chinese Medicine, Hubei University of Chinese Medicine, Xiangyang, 441000, China, hbtcm.edu.cn

**Keywords:** differentially expressed genes, feature genes, fibroblasts, hdWGCNA, machine learning, osteoarthritis

## Abstract

**Background:** Osteoarthritis (OA) is a degenerative joint disease with articular cartilage destruction, triggering a pro‐inflammatory response. The aim of this study was to screen key genes associated with fibroblasts based on single‐cell transcriptomic data and explore their potential value in OA diagnosis.

**Methods:** We obtained RNA sequencing (RNA‐seq) and single‐cell RNA‐seq (scRNA‐seq) data of OA from the Gene Expression Omnibus (GEO) database. The CellChat package for cell‐to‐cell communication analysis and identification of possible ligand‐receptor pairs. High‐dimensional weighted gene coexpression network analysis (hdWGCNA) was applied to identify the gene modules, and the key genes in the modules were identified and subjected to functional enrichment analysis. Subsequently, limma packages were used to screen for differentially expressed genes (DEGs) between OA and its control samples. Finally, the R package multipleROC was used to test the diagnostic potential of the screened key genes and to construct an OA diagnostic model using the rms package.

**Result:** Eight cell populations were identified and annotated based on scRNA‐seq and the percentage of fibroblasts was the highest. The cell–cell communication analysis has suggested that the highest communication probability was seen between mesenchymal cells/T cells and fibroblasts through the pairs of CD99‐CD99. The hdWGCNA analysis suggested that genes of modules M3, M4, M5, M6, and M8 (50 genes in total) were highly expressed in fibroblasts. Thereafter, we obtained 394 DEGs in OA and its control samples and took intersections with 50 modular genes and identified seven central genes (including apolipoprotein D *[APOD]*, biglycan *[BGN]*, *MXRA5*, *THY1*, *C1QTNF3*, dermatopontin [*DPT]*, and osteoglycin *[OGN]*). The constructed diagnostic models showed good predictive performance with all area under the curve (AUC) values >0.8. Finally, a satisfactory diagnostic model was established using these seven genes, and the differences in mRNA expression levels of these genes in OA and normal tissues were verified.

**Conclusion:** For the first time, our study systematically screened and validated key genes with diagnostic potential based on fibroblast‐specific single‐cell data in combination with hdWGCNA, providing a new theoretical basis and research direction for molecular typing and diagnosis of OA.

## 1. Introduction

Osteoarthritis (OA), recognized as the most common type of arthritis, is a comprehensive joint condition that results in significant pain and functional impairments, leading to notable morbidity [1, 2]. OA involves multiple anatomic and physiological alternations of joint tissues, including cartilage degradation, bone remodeling, and osteophyte formation, which results in the clinical manifestations including pain, stiffness, swelling, and joint function limitations [3–5]. Considering the high prevalence of OA around the world and no available causal treatment at present, it is therefore critical to reveal more reliable biomarkers for management in clinical practice [6, 7].

Despite the loss of articular cartilage being the hallmark characteristic of OA, the mechanisms underlying this OA‐related mechanism remain poorly understood. Synovium refers to a heterogeneous connective tissue exhibiting intrinsic pathologies during OA and aggravating the disease throughout the joint [8, 9]. Synovial fibroblasts (SFs) are a mixed stromal cell population possessing the capability of orchestrating inflammation or bone and cartilage damage [10, 11]. Moreover, SFs can be activated and differentiate into myofibroblast‐like cells, which have increased contractility and cause synovial fibrosis via depositing excess extracellular matrix (ECM) components [12]. In persistent diseases, fibroblasts can obtain an imprinted and aggressive phenotype, leading to the production of pro‐inflammatory cytokines and the ability to compromise tissue in the absence of continual stimuli [13]. Such an aggressive phenotype has offered attractive targets for therapeutics which could contribute to the alleviation of the burden of persistent inflammation.

Over the past decades, the rapid development of single‐cell transcriptomics has revolutionized the high‐resolution analysis of cellular composition [14], which has made it possible to better interpret the endogenous heterogeneity of cells in human OA cartilage [15]. Weighted gene coexpression network analysis (WGCNA) is an unbiased systems biology analysis method which aims to explore the coexpressed gene modules and identify core genes in the networks, whereas it can be applied for bulk RNA sequencing (RNA‐seq) [16, 17]. High‐dimensional WGCNA (hdWGCNA) is a comprehensive framework to analyze the coexpression networks in high‐dimensional transcriptomics data like single‐cell RNA‐seq (scRNA‐seq) and spatial RNA‐seq, which is able to perform isoform‐level network analysis with long‐read single‐cell data [18]. Prior investigations have delved into and fathomed out the aging‐related and immune‐associated feature biomarkers in OA [19, 20]. Nonetheless, present studies have not explored the efficacy of hdWGCNA in identifying the feature genes associated with fibroblasts in OA, which therefore permits us to bridge the gap in this study. In this in silico investigation, we coordinated the data of both scRNA‐seq and RNA‐seq via hdWGCNA, which, we hope, may contribute to the management of OA.

## 2. Methods

### 2.1. Data Source

In this study, we utilized transcriptomic data in order to screen for key genes and modeling of OA. The datasets GSE55235 and GSE55457 were downloaded from the Gene Expression Omnibus (GEO). GSE55235 included blood samples from 10 individuals with OA and 10 healthy controls. Similarly, GSE55457 comprised blood samples from 10 OA patients and 10 normal controls [21]. Both GSE55235 and GSE55457 were based on the GPL96 [HG‐U133A] Affymetrix Human Genome U133A Array. These two datasets were combined into a unified metadata cohort for integrated analysis due to their shared platform, which is crucial for data integration from various sources. The combat function within the “SVA” package was utilized to correct for batch effects. In addition, the GSE216651 dataset (including three OA samples) was used to assess the heterogeneity present in the different cell types of OA [22]. Information on the characteristics of the datasets used in this study is presented in Supporting Information Table S1.

### 2.2. Data Processing

For scRNA‐seq data, the “Seurat” R package was applied for the reading [23]. Cells with gene counts between 200 and 10,000 and with a mitochondrial gene ratio <10% were retained, and a total of 19,558 cells were obtained. Following the standardization via SCTransform and the dimensionality reduction via principal component analysis (PCA), the batch effects were removed via the “harmony” R package, followed by the dimensionality reduction via uniform manifold approximation and projection (UMAP) [24]. The first 20 principal components were applied for the construction of a K‐nearest neighbor (KNN) plot based on Euclidean distance using the “FindNeighbors” function. Cell populations were categorized thereafter via the “FindCluster” function at the resolution of 0.1 and annotated with the marker genes provided by the CellMarker2.0 databases.

### 2.3. Cell–cell Communication Analysis

The ligand–receptor pairs via which other cell populations affect fibroblasts were explored via “CellChat” R package [25, 26]. The communication possibility and significance (*p*‐value) of ligand‐receptor pairs between cell populations (which were set as “Cell–Cell Contact”) were demonstrated in bubble plots.

### 2.4. hdWGCNA

The rds data of RNA‐seq analysis was read via “hdWGCNA” R package [18]. Genes expressed in ≥5% of cells were first screened for analysis, and the network was built based on fibroblasts. The topological fit was evaluated by the “TestSoftPowers” function, and a soft threshold of *β* = 8 was chosen to construct the coexpression network. ConstructNetwork was used to build the coexpression network and score the gene‐to‐cell scores within the module, with the main parameters including “features = hMEs". Subsequently, the correlation between the modules and fibroblasts was calculated, and key genes were screened based on the connectivity (kME) of the genes in the modules for subsequent analysis.

### 2.5. Functional Enrichment Analysis

The key genes were uploaded to KOBAS‐i database (http://bioinfo.org/kobas) for the Gene Ontology (GO) and Kyoto Encyclopedia of Genes and Genomes (KEGG) functional enrichment analyses. The significantly enriched biological functions and functional pathways (*p*‐value < 0.05) were sorted out [27].

### 2.6. Differentially Expressed Genes (DEGs) Analysis

The “limma” R package was applied to calculate the difference on the gene expression level in control and OA groups [28, 29]. All *p*‐values were false discovery rate (FDR)‐corrected using the default Benjamini–Hochberg method of the limma package, with |log_2_fold change (FC)| ≥ 1 and adjusted. *p*.value < 0.01 as the final filtering criterion to ensure the robustness and reliability of the statistical results.

### 2.7. Construction of Diagnostic Model

The “multipleROC” R package (https://github.com/WandeRum/multiROC) was applied to test the diagnostic potential of the hub genes, following which the diagnostic model was constructed via “rms” R package and the nomogram was plotted. The relevant receiver operating characteristic (ROC) curve, calibration curve, and decision curve were drawn accordingly to evaluate the prediction potential and reliability of the diagnostic model.

### 2.8. Patients Samples

Synovial tissues were collected for this study from six individuals, who had total knee replacement surgery due to OA, as well as from six patients suffering from meniscus injuries, who had normal synovial tissues. Informed consent was obtained from all participants, and the samples were gathered, processed, and analyzed following the ethical guidelines established by the Ethics Committee at Xiangyang Hospital of Traditional Chinese Medicine, Hubei University of Chinese Medicine (Ethics Approval No. ZY2023Z016 and 2022BGE240).

### 2.9. Quantitative Real‐Time Polymerase Chain Reaction (qRT‐PCR)

Total RNA from the synovial tissue was purified using Trizol (Servicebio, Wuhan, China), and subsequently converted into complementary DNA (cDNA) through reverse transcription with Prime Script RT Master Mix (Takara, Shiga, Japan). For qRT‐PCR, we employed 2x SYBR Green qPCR Hub Mix (without ROX; Servicebio). The sequences of the primers for the seven essential genes are listed in Table 1. As an internal reference, the GAPDH gene was used.

**Table 1:** Primers applied in quantitative reverse‐transcription PCR.

Gene	Primers (5’‐3’)	
	Forward	Reverse
APOD	GAATCAAATCGAAGGTGAAGCCA	ACACGAGGGCATAGTTCTCAT
BGN	CAGTGGCTTTGAACCTGGAG	GGGAGGTCTTTGGGGATGC
MXRA5	TCAACGGCTTAACGTCTCTGA	CACGGATTTCCCTGCAAGTAAA
THY1	ATCGCTCTCCTGCTAACAGTC	CTCGTACTGGATGGGTGAACT
C1QTNF3	TCTCCACAAACCGGAGGACTA	CCTTGGTAGCCTCGAAAGC
DPT	GGGGCCAGTATGGCGATTATG	CGGTTCAAATTCACCCACCC
OGN	TCTACACTTCTCCTGTTACTGCT	GAGGTAATGGTGTTATTGCCTCA
GAPDH	GTCTCCTCTGACTTCAACAGCG	ACCACCCTGTTGCTGTAGCCAA

### 2.10. Statistical Analysis

All computational analysis was realized in R software (version 3.6.3, Irving Medical Center, Columbia University, New York, NY, USA). Experimental data were analyzed using GraphPad Prism software (version 8.0.2, GraphPad, Inc., La Jolla, CA, USA). The data were deemed to be statistically significant when the *p*‐value was below 0.05.

## 3. Results

### 3.1. Single‐Cell Landscape in OA

The single‐cell landscape in OA was depicted first to determine the cell population for our current research. Following the filtering, standardization, removal of batch effects, dimensionality reduction, eight main cell populations were identified and annotated based on their marker genes provided by CellMarker2.0 database and shown in brackets (Figure 1A,B): fibroblast (*DCN* and *LUM*), mesenchymal cell (*FN1* and *TIMP1*), endothelial cell (*VWF* and *PECAM1*), macrophage (*LYZ*, *C1QB* and *C1QA*), T cell (*CXCR4*, *CCL5* and *NKG7*), pericyte (*PDGFRB* and *RGS5*), B cells (*CD79A* and *CD27*), and transit amplifying cell (*TK1*, *MKI67* and *CCNB1*). The expression levels of these marker genes in the cell populations were also calculated and shown in Figure 1C. Further, we assessed the proportion of different cell types based on three case samples from the GSE216651 dataset and observed a larger proportion of fibroblast cell types in OA samples relative to other cell types (Figure 1D). These evidences, collectively, depicted the single‐cell landscape in OA and identified fibroblasts as the cell population for our current exploration.

Figure 1Single‐cell landscape in OA. (A) The single‐cell landscape in different datasets following the removal of batch effect. (B) UMAP plot on the distribution of different cell populations. (C) Expression levels of marker genes specific to the different cell populations. (D) Percentage of different cell populations in different datasets.

**Figure:**
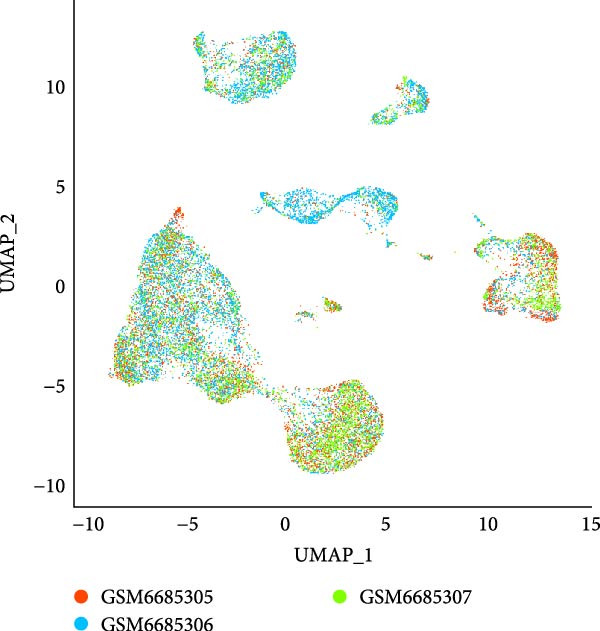
(A)

**Figure:**
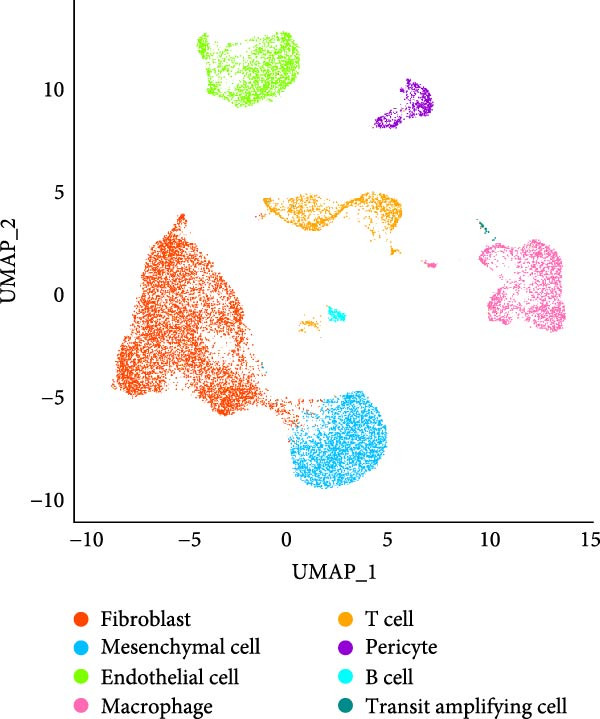
(B)

**Figure:**
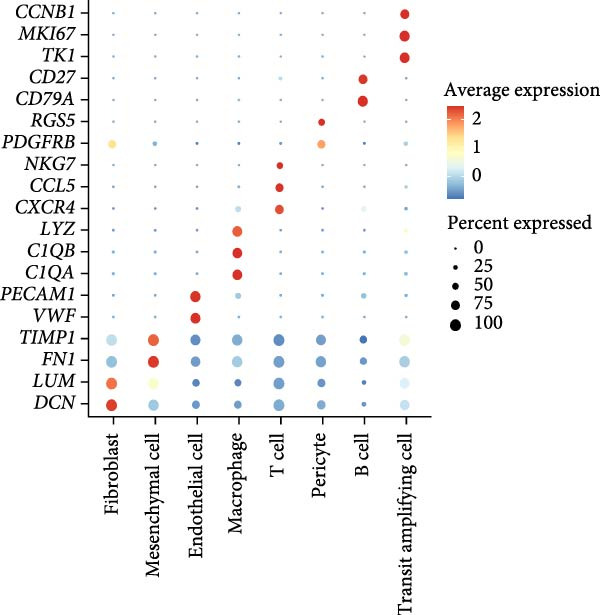
(C)

**Figure:**
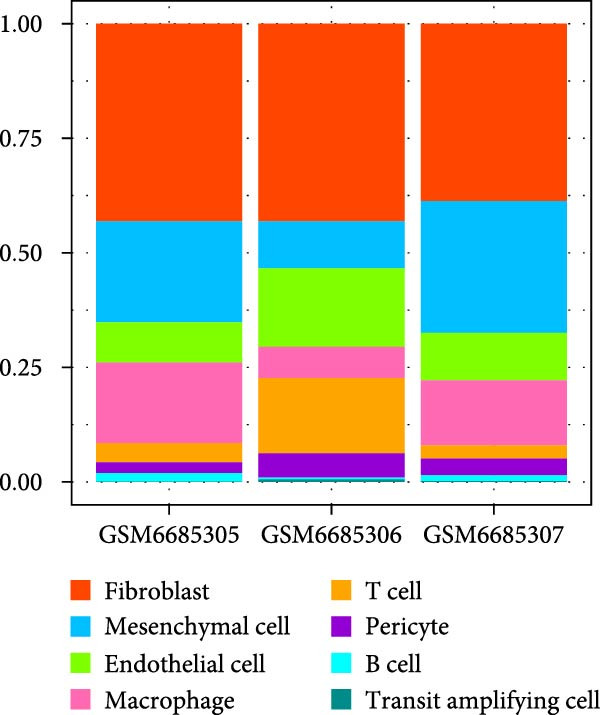
(D)

### 3.2. Cell–cell Communication Analysis

**Figure 2:**
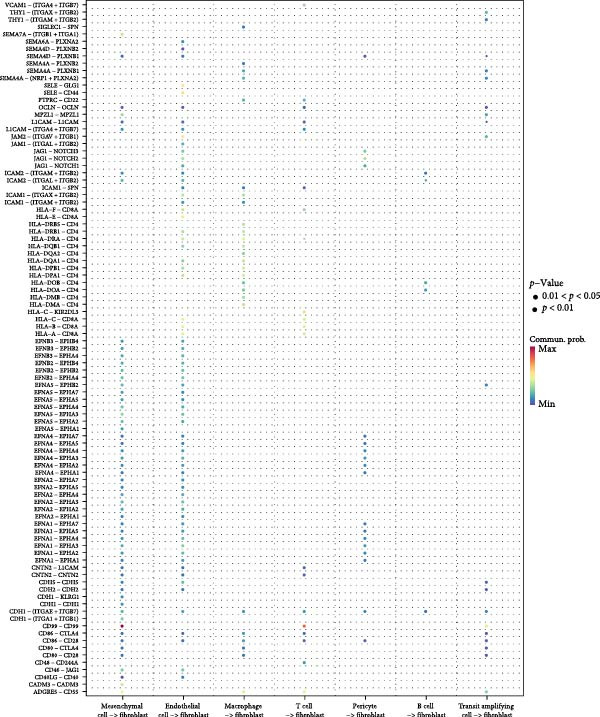
Bubble plots depicting the potential ligand‐receptor pairs via which fibroblasts and other cell population communicate with each other.

With the purpose of exploring the potential involvement and effects of fibroblasts in OA, cell–cell communication analysis was initiated to determine the ligand–receptor pairs via which fibroblasts communicate with other cell populations. A highest communication probability was seen between mesenchymal cells/T cells and fibroblasts through the pairs of CD99‐CD99 (Figure 2). The discoveries, accordingly, suggested that fibroblasts could indeed communicate with other cells to exert their effects on OA.

### 3.3. Identification of Fibroblasts‐Associated Gene Modules via hdWGCNA

Hereafter, we aimed to determine the feature genes in OA‐related fibroblasts, and the hdWGCNA analysis was accordingly carried out. The optimal soft threshold was calculated to be eight and the coexpression network was then established (Figure 3A,B). The connectivity of the gene module value and the module was calculated to sort the hub genes in the module and to divide the gene modules (from Fibroblasts‐M1 to Fibroblasts‐M10) (Figure 3C). The coexpression network of hub genes in each gene module was also illustrated in Figure 3D. Next, we calculated the genes specific to each module in different cell populations and found that in fibroblasts, only the genes of modules M3, M4, M5, M6, and M8 were highly expressed (Figure 3E). These modules were thereafter applied for subsequent analyses and the correlation matrix between these modules was also established (Figure 3F). Additionally, the hub gene network of these five modules was also depicted, where the 10 genes in the inner cycles were the key genes and the 15 genes in the outer cycles were the secondary key genes (Figure 3G). Finally, the single‐cell expression levels of hub genes specific to each gene module were shown in a UMAP plot (Figure 3H). In short, these findings highlighted the fibroblasts‐associated gene modules via hdWGCNA and paved the way for our succeeding analysis on the key fibroblasts‐associated genes in OA.

Figure 3Identification of fibroblasts‐associated gene modules via hdWGCNA. (A) Sorting process on the optimal soft threshold for hdWGCNA. (B) Coexpression network plot for hdWGCNA. (C) Division of gene modules for hdWGCNA. The *Y*‐axis represents the kME value and the gene list on the right was the hub genes specific to each gene module. (D) Coexpression network of hub genes specific to each gene module. (E) Expression levels of hub genes specific to each gene module in different cell populations. (F) The correlation matrix between these gene modules. (G) The hub gene network of these five modules. (H) The single‐cell expression levels of hub genes specific to each gene module.

**Figure:**
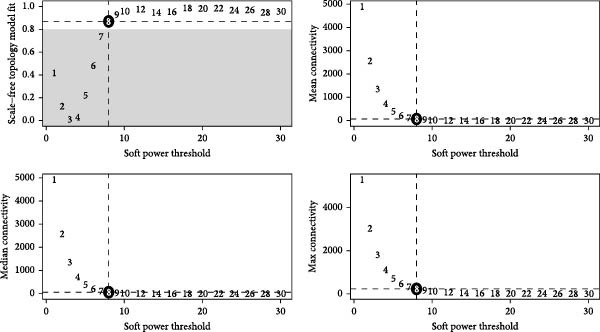
(A)

**Figure:**
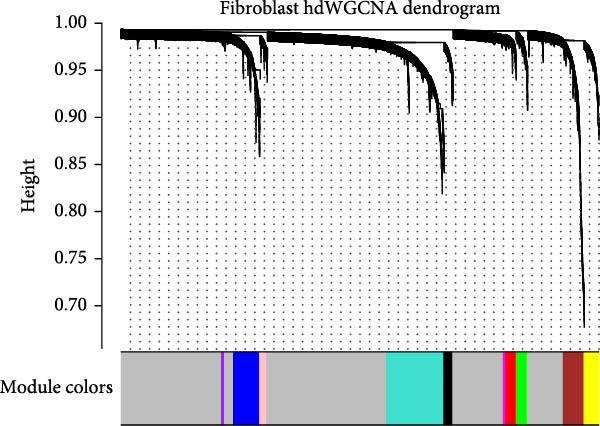
(B)

**Figure:**
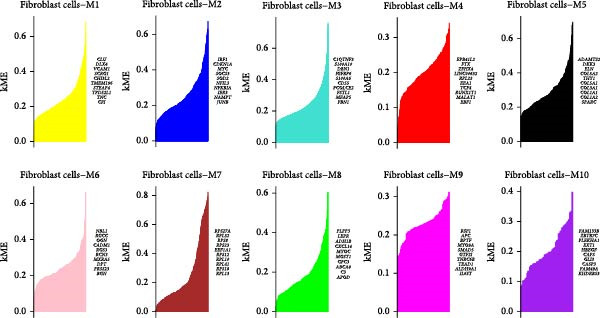
(C)

**Figure:**
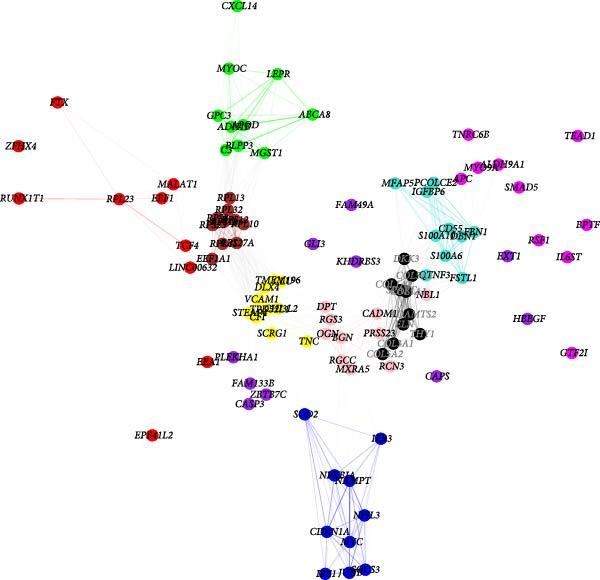
(D)

**Figure:**
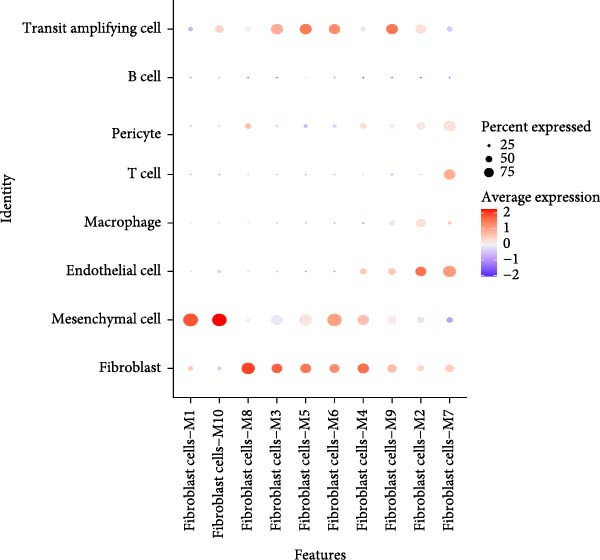
(E)

**Figure:**
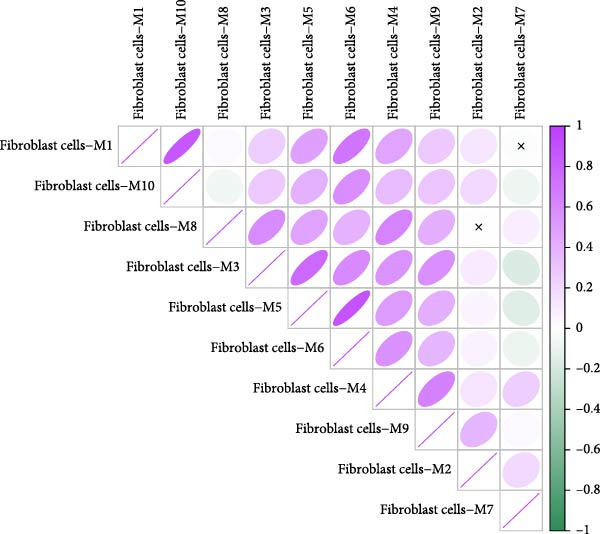
(F)

**Figure:**
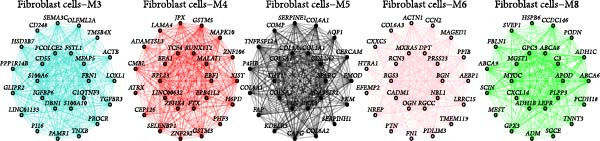
(G)

**Figure:**
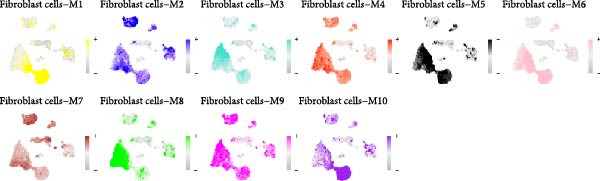
(H)

### 3.4. Functional Enrichment Analysis on Key Genes

Then the key genes were uploaded to the KOBAS‐i database to determine the enriched biological processes and functional pathways. The relevant results of GO enrichment analysis have suggested that these key genes were significantly enriched in collagen fibril organization, microfibril, and fibronectin binding (Figure 4A–C), and the results of KEGG enrichment analysis have revealed the significant enrichment of the key genes in protein digestion and absorption, the relaxin signaling pathway, and complement and coagulation cascade (Figure 4D). These discoveries depicted the enriched biological processes and functional pathways of the key genes in OA and aroused our interest to further explore the involvement of these key genes in OA.

Figure 4Functional enrichment analysis on key genes. (A–C) The results of GO functional enrichment analysis based on the biological process (A), cellular component (B) and molecular functions (C). (D) The results of KEGG enrichment analysis.

**Figure:**
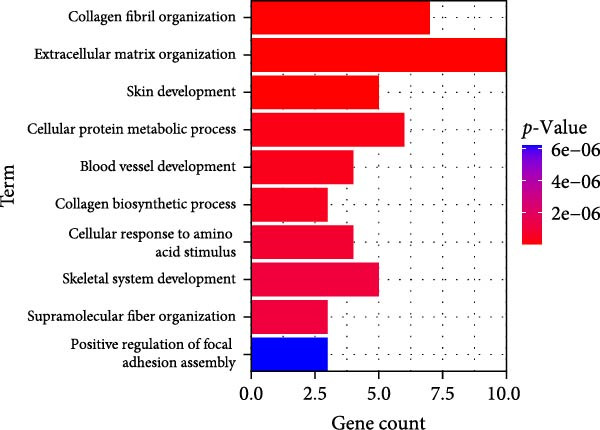
(A)

**Figure:**
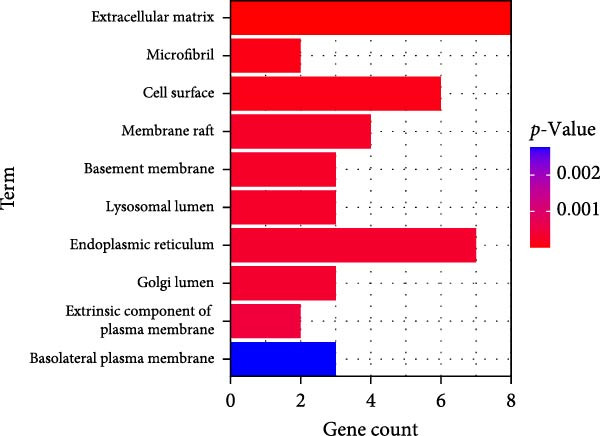
(B)

**Figure:**
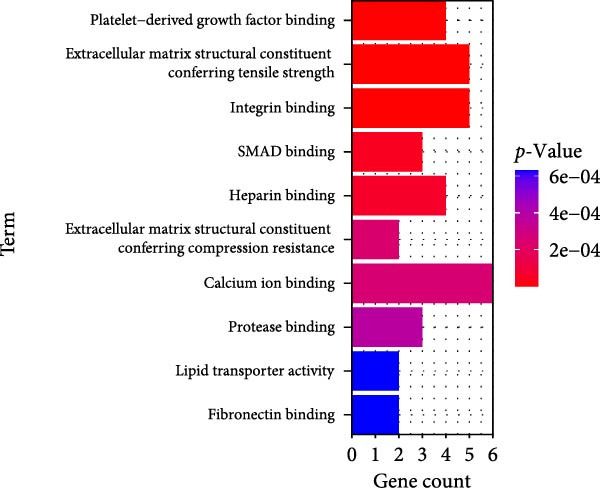
(C)

**Figure:**
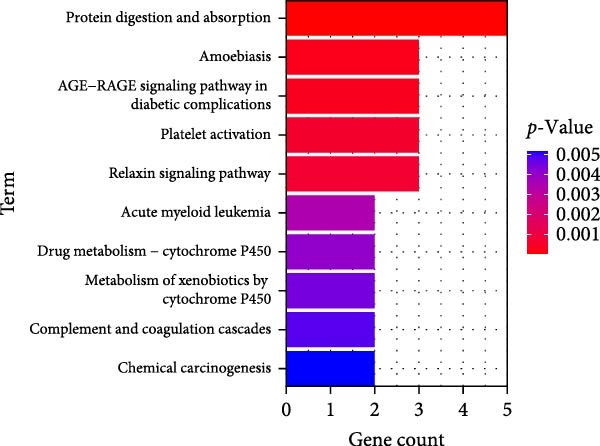
(D)

### 3.5. Differential Analysis on Sorting Fibroblasts‐Associated Hub Genes

The samples in the dataset were divided into OA and control groups, and the difference in the gene expression level was determined via “Limma” R package. The DEGs were finally sorted at the parameters |log_2_FC|≥1 and adjusted *p*‐value < 0.01. A total of 394 DEGs were identified and visualized in the volcano plot, including 210 upregulated genes and 184 downregulated genes (Figure 5A). In the end, we took the intersection of genes in the five key modules (50 genes) and DEGs (394 genes) to obtain seven key genes that met the dual criteria of “closely related to fibroblasts” and “significantly differentially expressed in OA,” namely, apolipoprotein D (*APOD*), biglycan (*BGN*), matrix remodeling associated 5 (*MXRA5*), Thy‐1 cell surface antigen (*THY1*), C1q and TNF related 3 (*C1QTNF3*), dermatopontin (*DPT*), and osteoglycin (*OGN*) (Figure 5B). Thereafter, the expression levels of these seven genes in both OA and control groups were illustrated in the heatmap (Figure 5C). In short, these evidences pointed out the fibroblast‐associated hub genes in OA, which is worthy of further exploration on their specific involvement in OA.

Figure 5Differential analysis on sorting fibroblasts‐associated hub genes. (A) Volcano lot on the differentially expressed genes. (B) Hubgene upset plot of differentially expressed genes and hdWGCNA‐related key genes. (C) Heatmap on the expression levels of seven hub genes in both control and OA groups.

**Figure:**
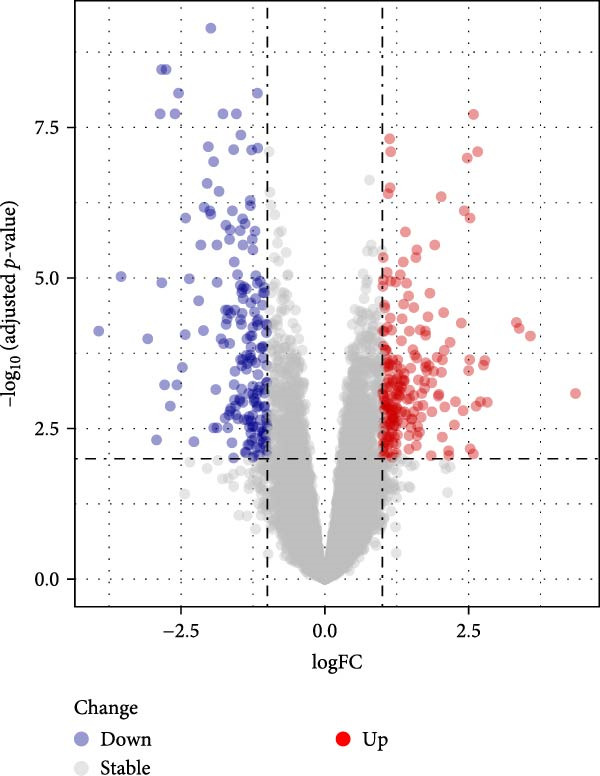
(A)

**Figure:**
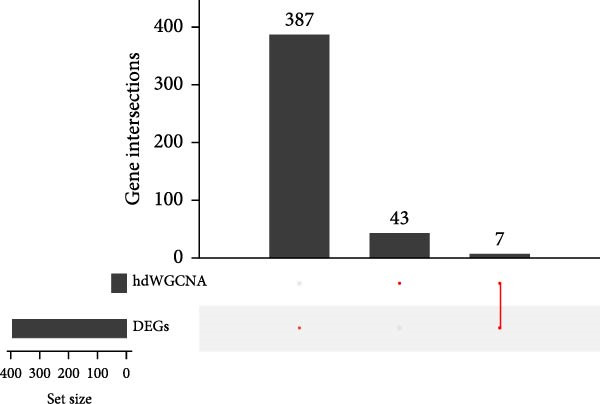
(B)

**Figure:**
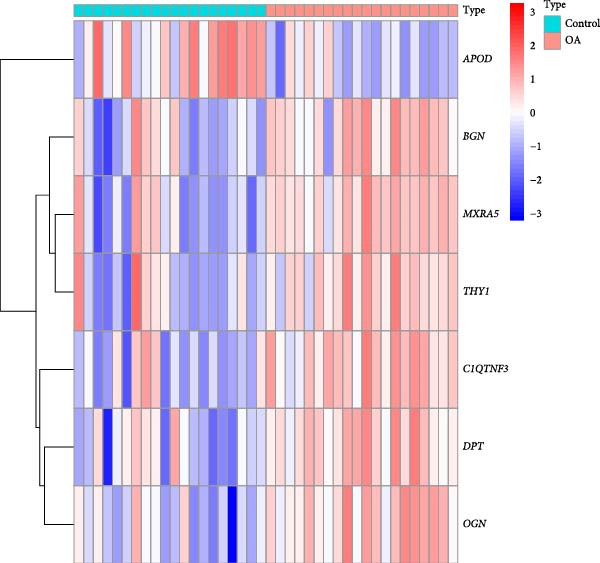
(C)

### 3.6. Construction of the Diagnostic Model

Thereafter, in order to determine the diagnostic efficacy of these seven genes in OA, the relevant ROC curves were plotted, and the area under the curve (AUC) was calculated as well. The AUC of these seven genes was all higher than 0.8, suggesting the satisfying diagnostic efficacy (Figure 6A, *p* < 0.001). A nomogram incorporating these seven genes was established then to construct the diagnostic model (Figure 6B), and the ROC curve and the corresponding AUC were also determined. The reliability of the diagnostic model was further confirmed (AUC: 0.962) (Figure 6C). The calibration curve is used to test the probabilistic consistency of model predictions, that is, the agreement between “predicted probability” and “observed probability.” This is particularly important for diagnostic models, especially if the predicted probabilities are to be used as further thresholds or to aid clinical judgment. As shown in Figure 6D, the ideal model should show a diagonal trend, that is, more accurate predictions. In addition, decision curve analysis (DCA) evaluates whether the model has a “net clinical benefit” under threshold probability, which is a practical guide to determine the clinical usability and practical value of the model.

Figure 6Construction of the diagnostic model. (A) ROC plot and the calculated AUC values of the seven hub genes. (B) The nomogram incorporating the seven hub genes. This nomogram shows a diagnostic model constructed based on seven genes, where the expression level of each variable corresponds to a certain score (Points), and the higher the total score, the higher the probability of predicting OA. The lower “Probability of Case” axis is used to translate the total score into the probability of OA risk. (C–E) The ROC curve (C), calibration curve (D), and decision curve (E) of the nomogram.

**Figure:**
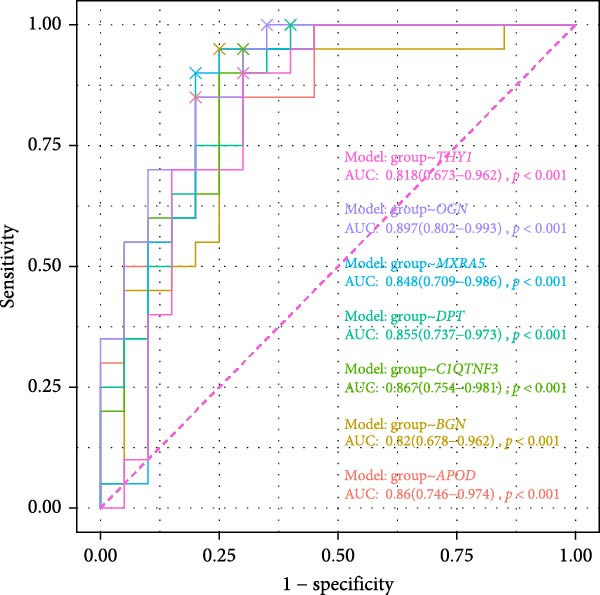
(A)

**Figure:**
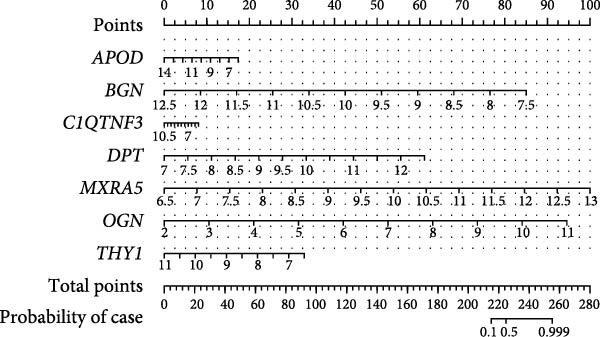
(B)

**Figure:**
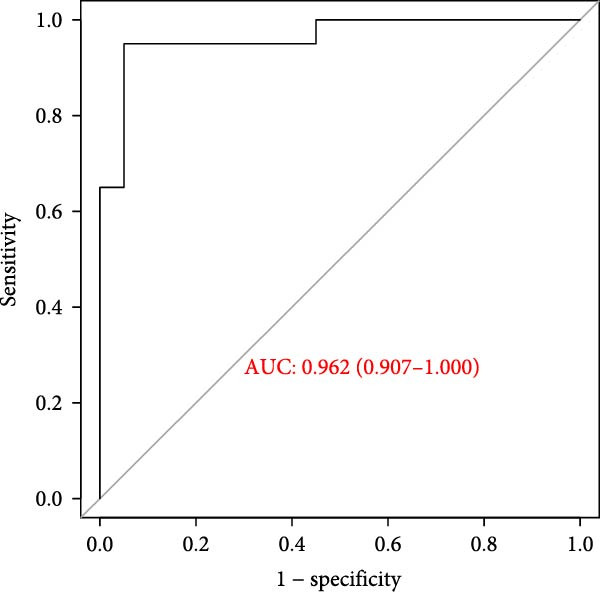
(C)

**Figure:**
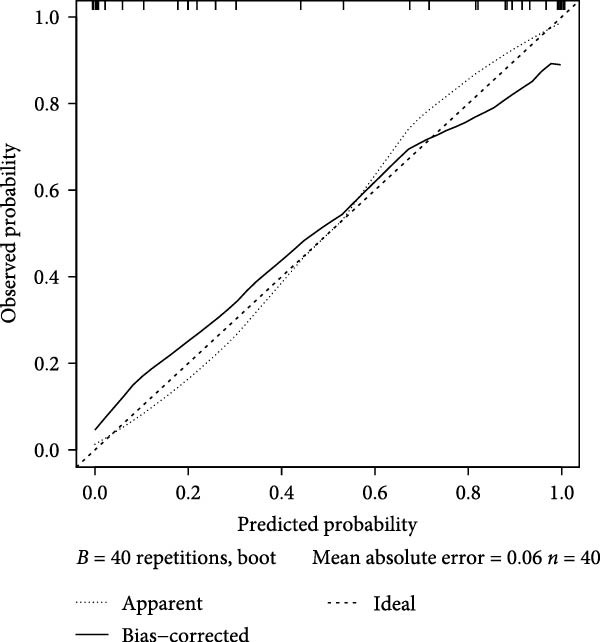
(D)

**Figure:**
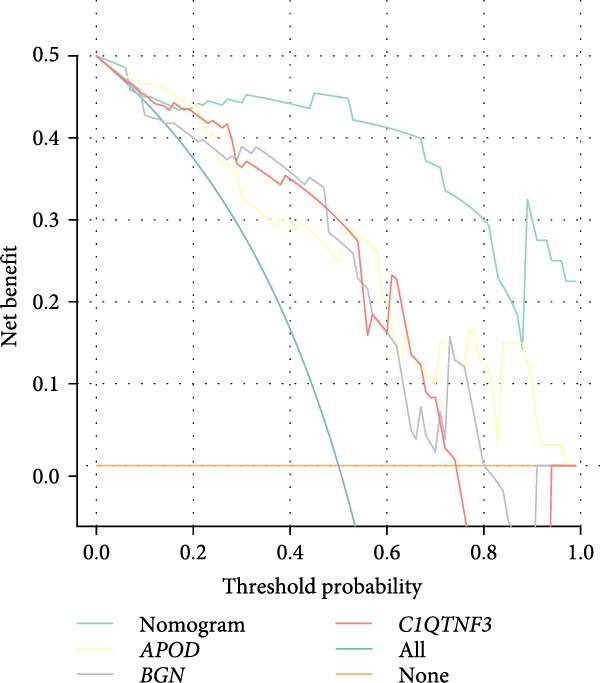
(E)

### 3.7. qRT‐PCR for Verification of Differential Expression of Seven Key Genes in OA Tissues

**Figure 7:**
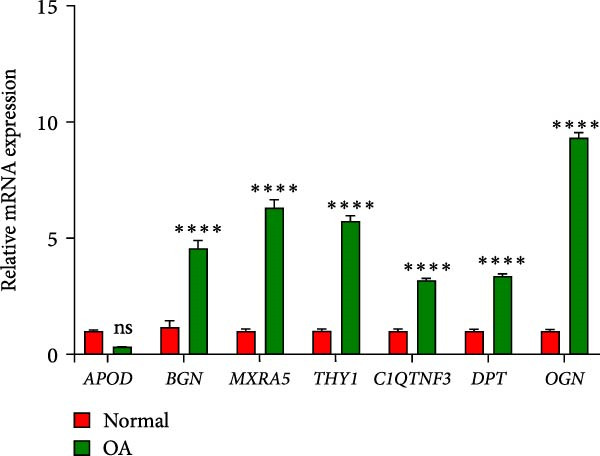
qRT‐PCR verified the expression levels of the screened genes (*APOD*, *BGN*, *MXRA5*, *THY1*, *C1QTNF3*, *DPT*, *OGN*) in OA and normal tissues.  ^∗∗∗∗^ means *p* < 0.001.

We further validated the differences in expression levels of the seven genes (*APOD*, *BGN*, *MXRA5*, *THY1*, *C1QTNF3*, *DPT*, *OGN*) screened in OA and its control tissues. As shown in Figure 7, we found that the expression levels of the six genes were significantly upregulated in OA tissues (*p* < 0.0001), except for *APOD*. These results further support the value of the study of these genes as potential diagnostic markers of fibroblast‐association in the development of OA.

## 4. Discussion

The genetics of OA has been transformed with the development and application of large‐scale genome‐wide association scans, and some techniques like statistical fine mapping, in silico analyses on genomics data, and laboratory‐based functional studies have contributed to the identification of some targets which can encode proteins with a variety of roles, including extracellular signaling molecules, intracellular enzymes, transcription factors (TFs), and cytoskeletal proteins [30]. So far, although the data from genetic studies in OA have not directly resulted in treatment, some OA‐associated genes code for proteins that have available therapeutics. Such genetic investigations have revealed some insights into OA and some options for translational intervention [30]. In recent years, there have indeed been many studies dedicated to mining OA‐related biomarkers. For example, Zhang et al. [31] identified four key markers (*CCR6*, *CLEC7A*, *IL18* and *SRSF2*) to be able to reliably differentiate between OA patients and healthy subjects using WGCNA, logistic regression and random forest (RF) models. Similarly, Chen et al. [32] went on to mine four OA centroids (*ZBTB16*, *TNFSF11*, *SCRG1*, and *KDELR3*) based on a combination of the WGCNA, Least Absolute Shrinkage and Selection Operator (LASSO), Support Vector Machine Recursive Feature Elimination (SVM‐RFE), and RF algorithms. In contrast, we conducted hdWGCNA for the first time based on fibroblast populations from single‐cell transcriptomic data, explicitly focusing on SFs, a cell type with important pathogenic potential in OA pathogenesis, thus enhancing the cell‐specificity and biological explanatory power of the screening results. Importantly, the seven key genes screened in this study are systematically presented for the first time as diagnostic factors specifically expressed in fibroblasts in the context of OA, which shows some novelty and specific localization value.

In our current study, we utilized multiple bioinformatics techniques to explore the feature biomarkers for OA. The scRNA‐seq data were applied first to reveal the cell population for our research. Based on the relevant data, fibroblasts were identified to be the cell population with the highest percentage in the GEO datasets. Fibroblasts are the mesenchymal cells involved in inflammatory diseases, which make up the stroma within the organ tissue and have been identified in existing scRNA‐seq analysis to be involved in OA [33, 34]. Thereafter, cell–cell communication analysis has revealed a close communication between mesenchymal cells/T cells and fibroblasts through the pairs of CD99‐CD99. CD99 is a cell surface antigen involved in crucial biological processes like cell adhesion, migration, differentiation, diapedesis and death, with influence on the inflammation‐related processes as well [35]. These findings reveal that fibroblasts in OA may communicate with other cell populations via CD99, which plays a potential impact on the inflammatory response in OA.

Previous studies applying WGCNA in the research of OA have revealed some potential therapeutic biomarkers in diagnosing OA [19, 20]. While there are no current investigations exploring the efficacy of hdWGCNA in identifying such biomarkers in OA, existing research has applied and discussed the efficacy of hdWGCNA in other diseases like ulcerative colitis, nonalcoholic fatty liver disease, and Crohn’s disease, to name a few [16, 36, 37]. Thereafter, our current study further expanded the application of hdWGCNA in identifying the feature genes in OA. In total 10 gene modules were accordingly revealed to be associated with fibroblasts in OA, five of which were shown to include genes with high expression in OA. These hdWGCNA‐related key genes were further applied for functional enrichment analysis, and it was clear that these genes were enriched in collagen fibril organization, microfibril, and fibronectin binding as well as protein digestion and absorption, relaxin signaling pathway, and the complement and coagulation cascade. Collagen fibril can assemble spontaneously from purified solutions of collagen molecules and their alternation leads to the pathological remodeling observed in OA [38, 39]. Fibronectin fragments, as the products of matrix degradation, can bind to some certain integrins and lead to the alternation of integrin signaling and the balance between intracellular anabolism and catabolism, thus aggravating the pathogenesis of OA [40]. Moreover, a prior exploration has suggested that relaxin can antagonize the SFs in OA, thereby preventing the flexion contracture of OA [41]. Complement, under the regulation of catabolic and inflammatory mediators, is shown to be implicated in OA‐relevant biological processes like ECM degradation, inflammatory response in chondrocytes and synoviocytes, and disbalanced bone remodeling, for example [42]. The specific involvement of these biological processes and functional pathways in OA, nonetheless, should be further explored.

Thereafter, with the purpose of exploring the fibroblasts‐associated hub genes for our research, the key genes of hdWGCNA analysis were intersected with the DEGs. A total of seven common genes were obtained, including *APOD*, *BGN*, *MXRA5*, *THY1*, *C1QTNF3*, *DPT*, and *OGN*. *APOD* is a glycoprotein first detected as a distinct component of the human plasma lipoprotein system, which acts on SFs to alleviate the process of OA in vitro [43, 44]. *BGN* is revealed to be affected by the stimulation of transforming growth factor‐β3 and epidermal growth factor (which is likely to contribute to the degenerative and regenerative effects observed in late stages of OA) [45]. *MXRA5* and *DPT* have been shown to exhibit an increased expression in OA based on a prior exploration on potential OA‐related biomarkers via WGCNA [46]. *THY1* was a cell surface antigen unveiled to be a diagnostic biomarker in OA as well [47]. *C1QTNF3* is an adipokine relative to in vitro chondrogenesis with chondroprotective effects in OA‐modeled SW1353 cells [48]. Besides, *OGN* is a class Ⅲ small leucine‐rich proteoglycan member involved in various physiological processes like collagen fibrillogenesis and correlated to the development and progression of OA based on another WGCNA analysis [49, 50]. A diagnostic model employing these seven genes was thereafter established with a satisfying predictive efficacy. The seven key fibroblast‐related genes we screened have been shown to be potentially associated with the pathological process of OA in previous studies, which, combined with the expression validation results of the present study, suggests their potential research value in the identification and mechanism studies of OA.

However, it should be noted that some research limitations exist in this study. First, the relatively small sample size of data integrated in this study, especially the single‐cell data derived from the tissues of only three OA patients, may not adequately reflect the overall heterogeneity of the OA patient population. For this reason, we plan to expand the sample size, especially to increase the samples of OA patients and controls from different regions, pathologic subtypes, and stages of disease progression, and to construct a larger validation cohort to improve the robustness and generalizability of the model. In addition, although we verified the expression trend of seven key genes by qRT‐PCR in multiple sample tissues, no protein‐level validation, or functional experiments were performed, which made it difficult to deeply illustrate the mechanism of these genes in OA. We will conduct further studies such as western blot, immunohistochemistry, and fibroblast intervention experiments to validate their functional roles in disease progression in combination with animal models of OA. Finally, this study focused on mining key markers in fibroblasts, and despite the uniqueness of this direction, OA is a complex disease with the involvement of multiple cell types, and focusing on a single cell type alone may limit a comprehensive understanding of its mechanisms. We plan to further analyze immune and structural cell subpopulations, such as macrophages, T cells, and chondrocytes in the synovium in conjunction with single‐cell data to explore their interactions with fibroblasts and to construct molecular network models at the multicellular level.

## 5. Conclusion

All in all, in this study, based on single‐cell transcriptome data with hdWGCNA, we systematically identified key gene modules closely related to fibroblasts and screened seven differentially expressed and highly related core genes (*APOD*, *BGN*, *MXRA5*, *THY1*, *C1QTNF3*, *DPT*, *OGN*). By constructing a diagnostic model and verifying by qRT‐PCR experiments, the expression of the above genes in OA tissues was confirmed, showing good diagnostic performance. The results of this study not only provide potential markers for molecular typing and diagnostic identification of OA but also lay the foundation for further revealing the role of fibroblasts in the pathogenesis of OA.

**Nomenclature**
APOD: Apolipoprotein D
AUC: Area under the curve
BGN: Biglycan
C1QTNF3: C1q and TNF related 3
DEGs: Differentially expressed genes
DPT: Dermatopontin
ECM: Extracellular matrix
FC: Fold change
GEO: Gene expression omnibus
GO: Gene ontology
hdWGCNA: High‐dimensional WGCNA
KEGG: Kyoto Encyclopedia of Genes and Genomes
KNN: K‐nearest neighbor
MXRA5: Matrix remodeling associated 5
OA: Osteoarthritis
OGN: Osteoglycin
PCA: Principal component analysis
RNA‐seq: RNA sequencing
ROC: Receiver operating characteristic
scRNA‐seq: Single‐cell RNA sequencing
SFs: Synovial fibroblasts
TFs: Transcription factors
THY1: Thy‐1 cell surface antigen
UMAP: Uniform manifold approximation and projection
WGCNA: Weighted gene coexpression network analysis.

## Ethics Statement

The authors have nothing to report.

## Consent

The authors have nothing to report.

## Disclosure

All authors read and approved the manuscript.

## Conflicts of Interest

The authors declare no conflicts of interest.

## Author Contributions

All authors contributed to this present work: Juan Xiao, Wei Lei, and Hao Zhang concepted and designed the research. Qunhai Wu, Feng Niu, and Honglin Pi acquired the data. Juan Xiao, Feng Niu, and Hao Zhang analyzed and interpreted data. Wei Lei, Juan Xiao, and Honglin Pi drafted the manuscript. Hao Zhang, Qunhai Wu, and Feng Niu revised manuscript for important intellectual content. Juan Xiao, Wei Lei, and Hao Zhang have contributed equally to this article.

## Funding

The study was funded by the Key Research and Development Plan of Xiangyang Science and Technology Department, 2014; Serial Number 7, the Key Research Project of Traditional Chinese Medicine Administration in Hubei Province (Grant ZY2023Z016), and the Hubei Provincial Central Government Guide Local Science and Technology Development Project (Grant 2022BGE240).

## Supporting Information

Additional supporting information can be found online in the Supporting Information section.

## Supporting information


**Supporting Information** Table S1. Characterization information of the dataset used for the study.

## Data Availability

The datasets generated and/or analyzed during the current study are available in the [GSE55235] repository, [https://www.ncbi.nlm.nih.gov/geo/query/acc.cgi?acc=GSE55235], [GSE55457] repository, [https://www.ncbi.nlm.nih.gov/geo/query/acc.cgi?acc=GSE55457] and [GSE216651] repository, [https://www.ncbi.nlm.nih.gov/geo/query/acc.cgi?acc=GSE216651].
