# Supplementary material for: Identification and Validation of Fibroblast‐Associated Genes in Osteoarthritis Based on High‐Dimensional Weighted Gene Coexpression Network Analysis
Source: J Immunol Res. 2025 Sep 28;2025:5547701. doi: 10.1155/jimr/5547701 (PMC12477407; doi:10.1155/jimr/5547701)
Supplement: Supplementary file 1 — Supporting Information Table S1. Characterization information of the dataset used for the study. [file JIMR-2025-5547701-s001.docx]

**Supplementary Table 1.** Characterization information of the dataset used for the study.

| Dataset | OA | Control | Platforms |
| --- | --- | --- | --- |
| GSE55235 | 10(synovial) | 10 (normal control synovial) | GPL96-57554 |
| GSE55457 | 10(synovial) | 10 (normal control synovial) | GPL96-57554 |
| GSE216651 | 3(synovium) |  | GPL24676 |
